# P-1086. COT-832, a Novel Polyene Macrolide Antifungal (I): *In Vitro* Antifungal Activity against *Candida*, *Aspergillus*, and *Mucor*, etc., Hemolytic Activity, and Cytotoxicity

**DOI:** 10.1093/ofid/ofae631.1274

**Published:** 2025-01-29

**Authors:** Hideki Maki, Yuri Nishiyama, Takafumi Ohara, Hideki Sugimoto

**Affiliations:** Shionogi & Co., Ltd., Toyonaka, Osaka, Japan; Shionogi & Co., Ltd., Toyonaka, Osaka, Japan; Shionogi & Co., Ltd., Toyonaka, Osaka, Japan; Shionogi & Co., Ltd., Toyonaka, Osaka, Japan

## Abstract

**Background:**

A novel polyene macrolide antifungal, COT-832 was created by SHIONOGI through structurally modifying Amphotericin B(AmB). Invasive fungal infections are commonly treated with antifungal drugs such as azoles, polyenes, and echinocandins; however, their efficacy is limited and mortality rates remain high. Therefore, the development of more effective and safer antifungal drugs is needed. AmB is available in formulations such as Fungizone (FGZ), which utilizes a deoxycholic acid formulation, and AmBisome (L-AMB), which employs a liposome formulation. While these polyene antifungals exhibit activity against a wide range of fungal species, their usage is often discontinued due to the nephrotoxicity associated with AmB itself. This study aimed to evaluate the antifungal activity, hemolytic activity, and cytotoxicity of COT-832 in vitro.

**Methods:**

Antifungal activity was assessed using the CLSI method on clinical isolates from Japan (2012-2016). Hemolysis activity was measured after applying compounds to 4% sheep red blood cells for 24 hours. Cytotoxicity was evaluated by applying compounds to HepG2 cells for 2 hours and measuring lactate dehydrogenase (LDH) levels in the supernatant with Cytotox96 assay for calculating cell survival rate.

**Results:**

Antifungal activity: COT-832 exhibited MIC90 of 0.5-2 µg/mL against Aspergillus and Candida, matching AmB's MIC. It also showed efficacy against azole-resistant A. fumigatus and echinocandin-resistant C. glabrata. COT-832's MIC90 for Cryptococcus was 0.5 µg/mL, surpassing AmB. Similar MICs were observed for rare molds.

Hemolytic activity: AmB caused hemolysis from 0.625 µg/mL, reaching 80% at 2.5 µg/mL. COT-832 displayed an 8% hemolysis rate at 2.5 µg/mL.

Cytotoxicity: AmB reduced cell count from 2 µg/mL, while no noticeable decrease occurred with COT-832 up to 72 µg/mL.

**Conclusion:**

COT-832 exhibits comparable antifungal activity to AmB while demonstrating reduced toxicity against various cells, up to a tenfold decrease compared to AmB. These findings suggest that COT-832 has potential for development as a safer polyene macrolide antifungal drug, offering potent antifungal activity across a wide range of fungal species.

**Disclosures:**

**Hideki Maki, PhD**, Shionogi & Co., Ltd.: employee|Shionogi & Co., Ltd.: employee **Yuri Nishiyama, MS**, Shionogi & Co., Ltd.: employee **Takafumi Ohara, n/a**, Shionogi & Co., Ltd.: employee **Hideki Sugimoto, n/a**, Shionogi & Co., Ltd.: employee

